# Effects of Acute Hypoxic Stress on Physiological and Hepatic Metabolic Responses of Triploid Rainbow Trout (*Oncorhynchus mykiss*)

**DOI:** 10.3389/fphys.2022.921709

**Published:** 2022-06-24

**Authors:** Buying Han, Yuqiong Meng, Haining Tian, Changzhong Li, Yaopeng Li, Caidan Gongbao, Wenyan Fan, Rui Ma

**Affiliations:** ^1^ State Key Laboratory of Plateau Ecology and Agriculture, Qinghai University, Xining, China; ^2^ College of Eco-Environmental Engineering, Qinghai University, Xining, China; ^3^ Qinghai Minze Longyangxia Ecological Aquaculture Co., Ltd., Longyangxia, China

**Keywords:** triploid rainbow trout, acute hypoxia, physiology, metabolism, HIF-2α

## Abstract

This experiment simulated the hypoxic environment caused by actual production operations in fish farming (i.e., catching, gathering, transferring, and weighting) to study the effects of acute hypoxic conditions on the physiological and metabolic responses of triploid rainbow trout (*O. mykiss*). Two groups of fish weighting 590 g were sampled in the normoxia group (dissolved oxygen above 7 mg/L) and hypoxia group (dissolved oxygen ranged from 2 to 5 mg/L for 10 min). The results showed that 1) regarding stress response, hypoxia increased plasma levels of cortisol, heat shock protein 70 (HSP-70), lysozyme, alanine aminotransferase (ALT), aspartate aminotransferase (AST) and creatine phosphokinase (CPK); induced the expression of hepatic genes encoding nuclear factor erythroid 2 related factor 2 (*Nrf2*), interferon *γ* (*IFN-γ*) and interleukin-1β (*IL-1β*). 2) Regarding metabolism response, hypoxia increased plasma levels of globulin (GLOB), glucose (GLU), triglyceride (TG) and lactate dehydrogenase (LDH); upregulated the hepatic gene expression of phosphoenolpyruvate carboxykinase, (*PEPCK*), pyruvate dehydrogenase kinase (*PDK1*), acetyl-CoA carboxylase (*ACC*) and acetyl-CoA oxidase (*ACO*); downregulated the hepatic gene expression of carnitine palmitoyl transferase 1 (*CPT1*); and unchanged the expression of hepatic genes in glycolysis and autophagy. 3) In response to hypoxia-inducible factors (HIFs), the hepatic *HIF-2α* gene was activated in the hypoxia group, but *HIF-1α* gene expression remained unchanged. Thus, during acute hypoxic stress, triploid rainbow trout were in a defensive state, with an enhanced immune response and altered antioxidant status. Additionally, the hepatic mitochondrial oxidation of glucose- and lipid-derived carbon in trout was suppressed, and hepatic gluconeogenesis and lipid synthesis were activated, which might be regulated by the HIF-2α pathway.

## Introduction

Rainbow trout (*O. mykiss*) is one of the most extensively farmed salmonid species, and in 2018, global production was over 848,000 tons (FAO, 2020). Because of its advantages of rapid growth, high meat quality and no gene pollution, triploid rainbow trout have become the main upmarket cold-water fish in China (Ma et al*.*, 2019). Dissolved oxygen (DO) is one of the main restricted environmental factors in fish farming. Rainbow trout have been shown to be a hypoxia-sensitive fish species when the DO in water is below 7 mg/L (Abdel-Tawwab et al*.*, 2019; Hou et al*.*, 2020). Triploid salmonid fish appear to require higher oxygen for growth and feeding than diploid fish (Hansen et al*.*, 2015). Therefore, hypoxic stress should be considered in triploid rainbow trout farming.

During the initial stage of hypoxia, some fish did not show any special activity and remained static at the bottom of the tank to conserve energy (Wu et al., 2007; Douxfils et al., 2012). With decreasing of DO levels, fish swam rapidly in a circular motion with a wide mouth opening, moved upward to the water surface, and began air breathing (Bowyer et al., 2015). A DO level consistently below 1–2 mg/L could lead to fish death (Abdel-Tawwab et al., 2019). Special production operations in fish farming, such as catching, gathering, transferring, or weighting, can cause local DO levels to decrease below 5 mg/L, or even 2 mg/L, within minutes. In land animals, the pulmonary vascular response was altered within 10 min of exposure to acute hypoxia in subjects developing high-altitude pulmonary edema (Dubowitz et al., 2009). In zebrafish, acute hypoxia (15 min at 10% or 5% air saturation) caused osmorespiratory compromise (Onukwufor and Wood, 2020). However, few studies have focused on the response of fish to short-term and intense hypoxic stress.

The effects of hypoxic stress on fish growth, physiological performance, immune responses and hypoxia signaling pathways have been reported (Zhu et al*.*, 2013; Xiao, 2015; Abdel-Tawwab et al., 2019). When DO is insufficient for the oxygen demands of fish, blood biochemical indicators reflecting fish physiological function are changed, such as the number of red blood cells; the levels of cortisol, triglyceride (TG), and glucose (GLU); and the activities of lactic dehydrogenase and transaminase (Barcellos et al*.*, 1999; Trenzado et al*.*, 2006; Martos-Sitcha et al*.*, 2019; Hou et al., 2020). Hypoxic stress can disturb the normal physiological function of fish by imposing oxidative stress on organisms by accelerating the generation of highly reactive oxygen species (ROS) (Lushchak, 2011). This causes oxidative damage and inflammatory reactions that seriously threaten the health of farmed fish. The aerobic metabolic pathway is converted into an anaerobic metabolic pathway to fulfill the high-energy requirements of fish during hypoxic stress (Abdel-Tawwab et al., 2019). Nutritional metabolism can be affected by hypoxia, but the results are controversial in different fish species and under different hypoxic conditions (acute or chronic stress) (Beck et al*.*, 2016; Pillet et al*.*, 2016; Li et al*.*, 2018). Based on the data from the terrestrial animals, adaptive changes in physiological and metabolic conditions in fish may be mediated by a family of hypoxia-inducible factors (HIFs) (Majmundar et al*.*, 2010; Mylonis et al*.*, 2019). Several studies have reported a connection between HIFs and hypoxia in Atlantic croaker (*Micropogonias undulatus*) (Rahman and Thomas, 2007), Eurasian perch (*Per*ca ﬂ*uviatilis*) (Rimoldi et al*.*, 2012), whitefish (*Coregonus clupeaformis*) (Whitehouse and Manzon, 2019), largemouth bass (*Micropterus salmoides*) (Yang et al*.*, 2019), and rainbow trout (Hou et al., 2020). However, the aforementioned information for triploid rainbow trout remains undefined.

Thus, this study simulated the hypoxic environment caused by actual production operations in fish farming to evaluate the effect of acute hypoxic stress on the physiological and metabolic responses of triploid rainbow trout by measuring plasma biochemical parameters, antioxidant capacity, immune responses, hypoxia-related gene expressions, and energy metabolism.

## Materials and Methods

### Fish, Feeding, and Sampling

This study was performed in strict accordance with the Standard Operation Procedures of the Guide for the Use of Experimental Animals of Qinghai University. The research protocol was reviewed and approved by the Ethical Committee of Qinghai University.

Female triploid rainbow trout from the same population were obtained from the Qinghai Minze Longyangxia Ecological Aquaculture Co., Ltd., China. Before the sampling, the water temperature was 14°C, and DO remained higher than 7 mg/L. Eighteen fish were randomly selected from a cultured cage and then sampled directly as the normoxic group. For the simulation of the hypoxic environment caused by actual production operations of catching and gathering in fish farming, 30 fish were randomly picked from the same culture cage and gently placed in a sealed plastic 400 L tank. When the DO in the tank was reached 5 mg/L because of fish breathing, the time was started. After hypoxic treatment for 10 min, DO decreased to 2 mg/L because of oxygen depletion, and some fish bellied up and floated in the water. Six seemingly normal fish were then sampled directly. The other new fish were picked from the same cage to repeat the aforementioned procedure twice. Eighteen fish were in the hypoxic group. DO and water temperature were continuously monitored using a DO meter (OxyGuard Handy Polaris portable, Denmark).

### Sample Collection

All sampled fish were anesthetized with eugenol (1:10,000) (Shanghai Reagent Corp, China) and then weighted (body weight, 592.89 ± 9.33 g; no difference was observed between the two groups). Blood was collected from the caudal vein by using heparinized syringes. Plasma was obtained by centrifugation at 3,000 rpm for 10 min. Plasma samples of similar volumes from three fish in the same group were pooled as one biological replicate. Liver tissue samples from the bled fish were subsequently dissected, and tissue samples of similar size were pooled according to the same procedure used for plasma. Six biological replicates (*n* = 6) from each group were analyzed. All samples were frozen in liquid nitrogen and then stored at −80°C for further biochemical and gene expression studies.

Liver samples were added to 0.9% cold physiological saline, and the ratio of tissue mass (g) to physiological saline (ml) was 1:9. Tissue homogenates (10%) were prepared by grinding with a tissue homogenizer (XHF-D, Xinzhi, China) and then homogenized by centrifugation at 4°C and 3,000 rpm for 10 min to determine the total protein, malondialdehyde (MDA), and total antioxidant capacity (T-AOC) contents.

### Biochemical Analysis

Plasma alanine aminotransferase (ALT), aspartate aminotransferase (AST), alkaline phosphatase (ALP), creatine phosphokinase (CPK), lactate dehydrogenase (LDH), total protein (TP), albumin (ALB), globulin (GLOB), glucose (GLU), triglyceride (TG), and total cholesterol (TC) levels were assayed in a certified hospital by using standard clinical methods in an automatic biochemical analyzer (ADVIA 2400; SIEMENS; Germany). Plasma cortisol, lysozyme, and heat shock protein 70 (HSP-70) levels were determined by enzyme-linked immunosorbent assay (ELISA), using commercial ELISA kits (Shanghai Enzyme Biotechnology Co., Ltd.).

The MDA and T-AOC contents in the plasma and liver were determined *via* a commercial kit (Nanjing Jiancheng Bioengineering Research Institute), using the TBA method and three FRAP methods, respectively. The protein content in the liver was determined by using a commercial kit (Nanjing Jiancheng Bioengineering Research Institute) and the Coomassie Brilliant Blue method.

### Total RNA Extraction and cDNA Synthesis

Total RNA was extracted from the liver tissue of triploid rainbow trout by using a total RNA extraction kit (DP419, Tiangen, China). A cDNA template was obtained using the PrimeScriptTM II1st strand cDNA Synthesis Kit (6210A, TaKaRa, Japan).

### Real-Time PCR Primer Design

According to the gene sequences of rainbow trout for nuclear factor erythroid 2 related factor 2 (*Nrf2*), superoxide dismutase (*SOD*), catalase (*CAT*), glutathione peroxidase (*GPx*), heme oxygenase 1 (*HO-1*), interferon *γ* (*IFN-γ*), tumor necrosis factor-α (*TNF-α*), heat shock protein 70 (*HSP-70*), interleukin-1β (*IL-1β*), interleukin-8 (*IL-8*), fatty acid synthase (*FAS*), acetyl-CoA carboxylase (*ACC*), acetyl-CoA oxidase (*ACO*), adipose differentiation-related protein (ADRP), carnitine palmitoyl transferase 1 (*CPT1*), pyruvate dehydrogenase kinase (*PDK1*), phosphoenolpyruvate carboxykinase, (*PEPCK*), glucose-6-phosphatase (*G6PASE*), glucokinase (*GK*), glucose transporter 2 (*GLUT2*), L-lactate dehydrogenase (*LDHA*), glycogen synthase (*GYS*), glycogen phosphorylase, liver form-like (*GYPL*), microtubule associated protein 1 light chain 3 beta (*LC3β*), autophagy-related 4β (*ATG4B*), autophagy-related 12 L (*ATG12L*), gamma aminobutyric acid receptor-associated protein (*GABARAPL1*), hypoxia-inducible factor 1α (*HIF-1α*), hypoxia-inducible factor 2α (*HIF-2α*), factor inhibiting hypoxia-inducible factor 1(*FIH1*), and egl nine 1-like protein (*EGLN1*/*PHD2*), real-time PCR primers were designed using Primer 6.0 software. The primer sequences are shown in Table 1.

**TABLE 1:** Primers used for gene expression analysis in *Oncorhynchus mykiss*.

Primer	Primer sequence (5′to 3′)	Gene	Accession
Hypoxia-related genes			
RTHIF-1α-F1	TCT​GAG​GAC​GGG​GAC​ATG​AT	*HIF-1α*	AF304864.1
RTHIF-1α-R1	GGT​CTG​AGC​AGT​GGA​GAA​CC		
RTHIF-2α-F1	GGT​TAC​ATC​AGA​CGG​CGA​CA	*HIF-2α*	XM_021576379.1
RTHIF-2α-R1	CCT​TCT​TCC​CAG​TGC​CAT​TTT		
RTFIH1-F1	ACA​GCC​CTA​TCT​GGA​ACG​ACT​C	*FIH1*	NM_001281328.1
RTFIH1-R1	CCA​CTG​GTT​GCT​CGT​TGT​TTA​T		
RTPHD2-F1	TGG​AAA​ACC​TGC​TTA​AAT​GTG​GAC	*PHD2*	HQ615594.1
RTPHD2-R1	TTT​GAA​CCG​CTT​GCC​TTG​C		
Antioxidant-related genes			
RTNrf2-F1	GCA​GAG​GTC​TGC​CCA​CCT​GAA​T	*Nrf2*	HQ916348.1
RTNrf2-R1	GCC​ACA​AGG​CAG​GGT​GAC​ACT​T		
RTSOD-F1	TGA​AGG​CTG​TTT​GCG​TGC​TGA​C	*SOD*	NM_001160614.1
RTSOD-R1	CCG​TTG​GTG​TTG​TCT​CCG​AAG​G		
RTCAT-F1	CCG​TCC​TTC​GTC​CAC​TCT​CAG​A	*CAT*	XM_021568213.1
RTCAT-R1	CTC​GGC​ATC​CTC​AGG​CTT​CAA​G		
RTGPx-F1	TCA​TCA​TGT​GGA​GCC​CTG​TCT​G	*GPx*	AF281338.1
RTGPx-R1	TCT​GCC​TCA​ATG​TCA​CTG​GTC​A		
RTHO-1-F1	CGC​CTA​CAC​CCG​TTA​CCT​AG	*HO-1*	XM_021558210.1
RTHO-1-R1	CTC​TCC​GCT​GCT​TAA​CCC​AA		
Immune-related genes			
RTIFN-γ-F1	TAC​CCT​CAC​CTT​CCC​ACC​A	*IFN-γ*	NM_001124620.1
RTIFN-γ-R1	TTC​CTG​CGG​TTG​TCC​TTC​TT		
RTTNF-α-F1	GGC​GAG​CAT​ACC​ACT​CCT​CTG​A	*TNF-α*	AJ401377.1
RTTNF-α-R1	AGC​TGG​AAC​ACT​GCA​CCA​AGG​T		
RTHSP-70-F1	GGA​CGC​AGC​CAA​GAA​CCA​AGT	*HSP-70*	AB062281.1
RTHSP-70-R1	GGC​CGT​GTC​GAG​TCG​TTG​AT		
RTIL-1β-F1	ACG​GTT​CGC​TTC​CTC​TTC​TAC​A	*IL-1β*	AJ557021.2
RTIL-1β-R1	GCT​CCA​GTG​AGG​TGC​TGA​TGA​A		
RTIL-8-F1	GTC​AGC​CAG​CCT​TGT​CGT​TGT	*IL-8*	NM_001124362.1
RTIL-8-R1	CGT​CTG​CTT​TCC​GTC​TCA​ATG​C		
Glycometabolism genes			
RTGK-F1	AGA​TCA​CTG​TGG​GCA​TCG​AC	*GK*	AF053331.2
RTGK-R1	GAT​GTC​ACA​GTG​AGG​CGT​CA		
RTLDHA-F1	GGC​GTG​AAT​GTT​GCT​GGT​GT	*LDHA*	XM_021564046.1
RTLDHA-R1	TCC​TCC​TTG​TCT​GCG​TCT​GTG		
RTPEPCK-F1	CGG​TGT​GTT​TGT​AGG​AGC​CT	*PEPCK*	NM_001124275.1
RTPEPCK-R1	ACG​TGG​AAG​ATC​TTG​GGC​AG		
RTG6PASE-F1	GCT​GAC​CTG​CAT​ACC​ACC​TT	*G6PASE*	XM_021575943.1
RTG6PASE-R1	CAG​CCA​CCC​AGA​TGA​GCT​TT		
RTPDK1-F1	CAGACCCCATCGTCAGCC	*PDK1*	XM_021597164.1
RTPDK1-R1	TAC​CCT​CAC​CTT​CCC​ACC​A		
RTGLUT2-F1	GGA​CCA​GCA​ACT​TCA​TCA​TAG​GC	*GLUT2*	AF321816.1
RTGLUT2-R1	CCA​AAC​AAC​AGC​ACA​GCA​AAC​A		
RTGYS-F1	GAC​AGA​GAG​GCC​AAC​GAC​TC	*GYS*	XM_021563420.1
RTGYS-R1	ACT​CAT​GGA​AAT​GGG​CGA​GG		
RTGYPL-F1	TGA​TTA​ACC​TGG​GGC​TGC​AG	*GYPL*	XM_021585435.1
RTGYPL-R1	GCC​ATC​GAG​TCC​AGG​AAA​CA		
Lipometabolism genes			
RTFAS-F1	TCT​AGA​GAC​GCC​ACC​TTC​GA	*FAS*	XM_021581290.1
RTFAS-R1	TGC​AGT​TTC​TCC​TCA​GCC​AG		
RTACC-F1	TCA​TCA​ATG​CCA​AGG​ACC​CC	*ACC*	XM_021618451.1
RTACC-R1	CGT​CAG​AGT​CCA​GGT​TTG​CT		
RTCPT1-F1	TAC​AGC​TGG​CCC​AAT​TCA​GG	*CPT1*	AF327058.3
RTCPT1-R1	TCG​CAG​TGT​TCT​TGT​CCT​CC		
RTACO-F1	TTG​GGC​CTC​ATC​ATT​GCA​GT	*ACO*	XM_021613038.1
RTACO-R1	ACT​GGG​TCT​GGT​GCT​CAA​TG		
RTADRP-F1	CAG​ATG​GTC​AGC​AGC​GGA​ATG	*ADRP*	XM_021615225.1
RTADRP-R1	GAG​CCC​AGA​CGG​ACA​TAG​TAG​C		
Autophagy-related genes			
RTLC3β-F1	CCC​CAA​CAA​GAT​TCC​GGT​CA	*LC3β*	KX845472.1
RTLC3β-R1	GGT​TGG​AGT​TCA​GCT​GGA​GG		
RTGBRAP-F1	TAC​CTT​GTG​CCC​TCT​GAC​CT	*GABARAPL1*	NM_001165091.1
RTGBRAP-R1	GCT​GAG​GTG​GGA​GGA​ATG​AC		
RTATG4B-F1	TAT​GCG​CTT​CCG​AAA​GTT​GTC	*ATG4B*	CA345181.1
RTATG4B-R1	CAG​GAT​CGT​TGG​GGT​TCT​GC		
RTATG12L-F1	TGG​AGG​CCA​ATG​AAC​AGC​TG	*ATG12L*	XM_021623074.1
RTATG12L-R1	CTT​CCC​ATC​GCT​GCC​AAA​AC		
Reference gene			
RTβ-actin-F1	TAC​AAC​GAG​CTG​AGG​GTG​GC	*β-actin*	AJ438158.1
RTβ-actin-R1	GGC​AGG​GGT​GTT​GAA​GGT​CT		

### Gene Expression Analysis

Real-time PCR was conducted using a PCR instrument (Light Cycler^®^ 480, Roche, United States) in a final volume of 10 μl containing 5 μl SYBR^®^ Premix Ex TaqTM II (2×), 0.3 μl forward primer, 0.3 μl reverse primer, 1 μl cDNA template, and 3.4 μl sterile water. The thermal cycling system reaction conditions were 95°C for 5 min; 95°C for 30 s, 60°C for 30 s, and 72°C for 30 s; and 40 cycles of 72°C, for 10 min. Each reaction was repeated three times. The relative abundance of the genes was normalized to that of *β-actin* and calculated using the 2^−ΔΔCt^ method.

### Statistical Analysis

Statistical analysis of the experimental data was performed using SPSS 20 data analysis software, and the obtained data were expressed as the mean ± standard error. The effects of normoxia and hypoxia on individual indicators were analyzed using independent samples *t-*test. Compared with the normal group, *p < 0.05; **p < 0.01.

## Results

### Effect of Acute Hypoxic Stress on the Plasma Biochemical Parameters of Triploid Rainbow Trout

As shown in Table 2, the ALT, AST, LDH, CPK, GLOB, GLU, and TG levels were significantly higher in the hypoxic group, but ALB level was lower than that in the normoxic group (*p* < 0.05). Particularly notable is that the maximal increase in LDH level was approximately 4.6-fold higher in the hypoxic group than in the normoxic group. No significant differences were observed in the plasma TP, TC, and ALP levels between the two groups (*p* > 0.05).

**TABLE 2:** Effect of acute hypoxic stress on the plasma biochemical parameters of triploid rainbow trout (*n* = 6).

Biochemical parameters	Normoxic group	Hypoxic group	*t*-test
ALT^a^ (U/L)	11.40 ± 0.40	21.80 ± 1.80	**
AST^b^ (U/L)	276 ± 13.40	553 ± 42.00	**
ALP^c^ (U/L)	186 ± 7.94	207 ± 16.50	ns
CPK^d^ (U/L)	3414 ± 321	6688 ± 228	**
LDH^e^ (U/L)	213 ± 13.60	982 ± 135.00	**
TP^f^ (g/L)	42.90 ± 0.71	43.10 ± 0.95	ns
ALB^g^ (g/L)	16.10 ± 0.25	15.10 ± 0.34	*
GLOB^h^ (g/L)	26.70 ± 0.54	28.30 ± 0.56	*
GLU^i^ (mg/dl)	91.08 ± 6.66	127.80 ± 3.60	**
TC^j^ (mg/dl)	425.37 ± 11.99	444.71 ± 21.66	ns
TG^k^ (mg/dl)	383.66 ± 8.84	472.94 ± 15.03	**
Cortisol (pg/ml)	325.75 ± 6.26	426.75 ± 11.64	**
HSP-70^l^ (pg/ml)	115.41 ± 3.35	132.39 ± 3.97	**
Lysozyme (U/L)	1.69 ± 0.04	2.01 ± 0.05	**

Note: mean ± standard error; ns, no significant difference (*p* > 0.05); *p < 0.05; **p < 0.01.

a ALT, alanine aminotransferase.

b AST, aspartate aminotransferase.

c ALP, alkaline phosphatase.

d CPK, creatine phosphokinase.

e LDH, lactic dehydrogenase.

f TP, total protein.

g ALB, albumin.

h GLOB, globulin.

i GLU, glucose.

j TC, total cholesterol.

k TG, triglyceride.

l HSP-70, heat shock protein 70.

Under hypoxic conditions, plasma levels of lysozyme, cortisol, and HSP-70 increased significantly (*p* < 0.01; Table 2).

### Effect of Acute Hypoxic Stress on the Hypoxia-Related Gene Expression in the Liver of Triploid Rainbow Trout

**FIGURE 1:**
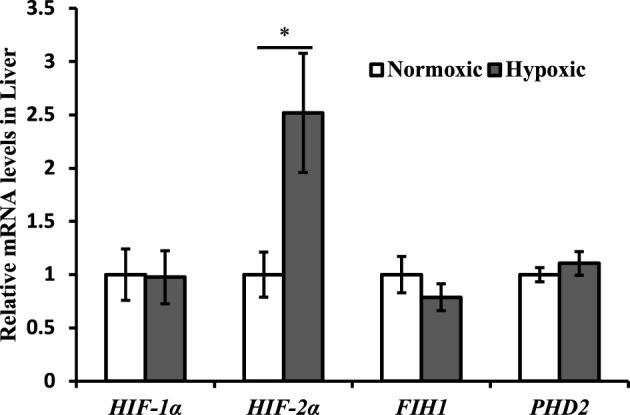
Effect of acute hypoxic stress on the expression of the hypoxia-related genes in the liver of triploid rainbow trout (*n* = 6; *HIF-1α*, hypoxia-inducible factor 1α; *HIF-2α*, hypoxia-inducible factor 2α; *FIH1*, factor inhibiting hypoxia-inducible factor 1; *PHD2*, egl nine 1-like protein). Asterisks indicate the significant difference (*t*-test; *p < 0.05; **p < 0.01).

Changes in the expression of hypoxia-related genes are shown in Figure 1. The mRNA expression of *HIF-2α* was upregulated in the liver of triploid rainbow trout under acute hypoxic stress (*p* < 0.05; Figure 1). Particularly notable is that the maximal increase in *HIF-2α* was approximately 2.5-fold higher in the hypoxic group than in the normoxic group. However, the mRNA expression of *HIF-1α, FIH1, and PHD2* was not affected by acute hypoxic stress (*p* > 0.05).

### Effect of Acute Hypoxic Stress on the Antioxidant Capacity and Antioxidant Related Genes Expressions in the Plasma or Liver of Triploid Rainbow Trout

**FIGURE 2:**
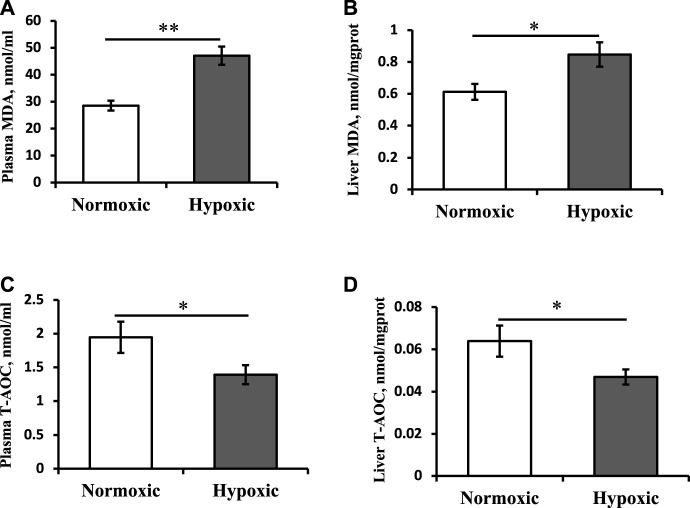
Effect of acute hypoxic stress on the malondialdehyde (MDA) content in the plasma **(A)** and liver **(B)** as well as the total antioxidant capacity (T-AOC) level in the plasma **(C)** and liver **(D)** of triploid rainbow trout (*n* = 6). Asterisks indicate the significant differences (*t*-test; *p < 0.05; **p < 0.01).

As shown in Figure 2, the MDA contents of the plasma and liver in the hypoxic group were significantly higher than those in the normoxic group (Figures 2A,B; *p* < 0.05). T-AOC levels in the plasma and liver showed trends opposite those of MDA content (Figures 2C,D; *p* < 0.05).

**FIGURE 3:**
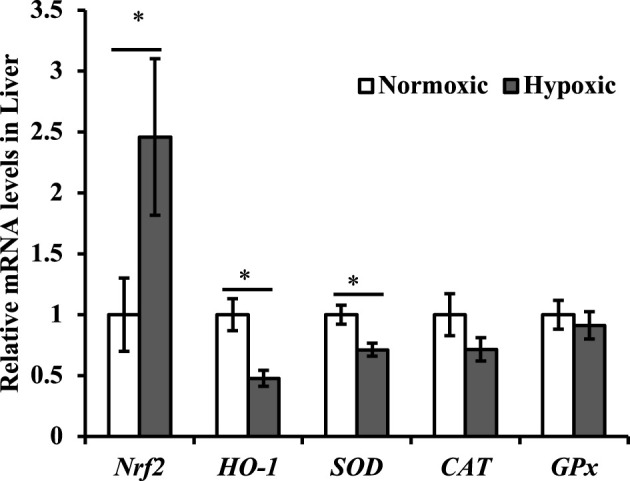
Effect of acute hypoxic stress on the expression of the antioxidant-related genes in the liver of triploid rainbow trout (*n* = 6; Nrf2, nuclear factor erythroid 2 related factor 2; SOD, superoxide dismutase; CAT, catalase; GPx, glutathione peroxidase). Asterisks indicate significant differences (*t*-test; *p < 0.05; **p < 0.01).

The effect of acute hypoxic stress on the antioxidant gene expression in the liver is shown in Figure 3. The expression of *Nrf2* mRNA was upregulated in the liver, and the mRNA expression of *SOD* and *HO-1* was downregulated (*p* < 0.05; Figure 3). Particularly notable is that the maximal increase in *Nrf2* was approximately 2.5-fold higher in the hypoxic group than in the normoxic group. No significant difference was observed in the mRNA expression of *CAT* and *GPx* in the liver of triploid rainbow trout (*p* > 0.05).

### Effect of Acute Hypoxic Stress on the Immune-Related Genes Expressions in the Liver of Triploid Rainbow Trout

**FIGURE 4:**
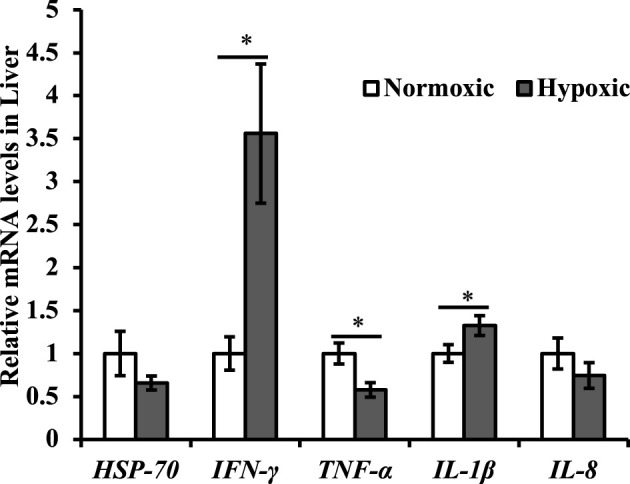
Effect of acute hypoxic stress on the expression of the immune-related genes in the liver of triploid rainbow trout (*n* = 6; IFN-γ, interferon γ; TNF-α, tumor necrosis factor-α; HSP-70, heat shock protein 70; IL-1β, interleukin-1β; IL-8, interleukin-8). Asterisks indicate significant differences (*t*-test; *p < 0.05; **p < 0.01).

Changes in the expression of genes related to the immune system are shown in Figure 4. The mRNA expression of *IFN-γ* and *IL-1β* in the liver were upregulated under acute hypoxic stress (*p* < 0.05; Figure 4). Additionally, the maximal increase in *IFN-γ* was approximately 3.5-fold higher in the hypoxic group than in the normoxic group. However, the mRNA expression of *TNF-α* in the liver was downregulated (*p* < 0.05; Figure 4). No significant differences were observed in the mRNA expression levels of *HSP-70* and *IL-8* in the liver of triploid rainbow trout (*p* > 0.05; Figure 4).

### Effect of Acute Hypoxic Stress on the Glycometabolic Genes Expressions in the Liver of Triploid Rainbow Trout

**FIGURE 5:**
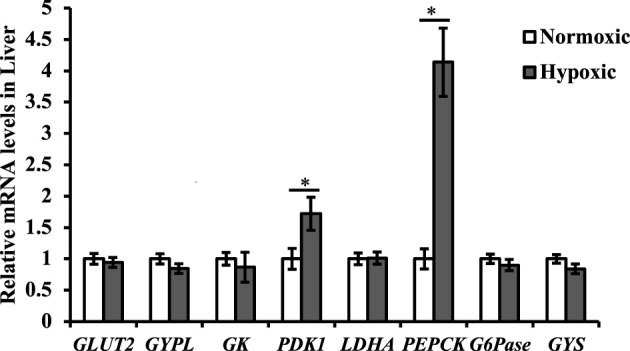
Effect of acute hypoxic stress on the expression of the glycometabolic genes in the liver of triploid rainbow trout (*n* = 6; GLUT2, Glucose transporter 2; GYPL, Glycogen phosphorylase liver form-like; GK, Glucokinase; PDK1, Pyruvate dehydrogenase kinase; LDHA, L-lactate dehydrogenase; PEPCK, Phosphoenolpyruvate carboxykinase; G6Pase, Glucose-6-phosphatase; GYS, Glycogen synthase). Asterisks indicate significant differences (*t*-test; *p < 0.05, **p < 0.01).

Data on the mRNA expression of the glycometabolic genes are shown in Figure 5. The mRNA expression of *PEPCK* and *PDK1* in liver tissue was upregulated under acute hypoxic stress (*p* < 0.05). Additionally, the maximal increase in *PEPCK* was approximately 4-fold higher in the hypoxic group than in the normoxic group. However, no significant differences were observed in the mRNA expression levels of *GLUT2*, *GYPL, GK*, *LDHA*, *G6Pase,* and *GYS* in the liver of triploid rainbow trout under acute hypoxic stress (*p* > 0.05).

### Effects of Acute Hypoxic Stress on the Lipometabolic Gene Expression in the Liver of Triploid Rainbow Trout

**FIGURE 6:**
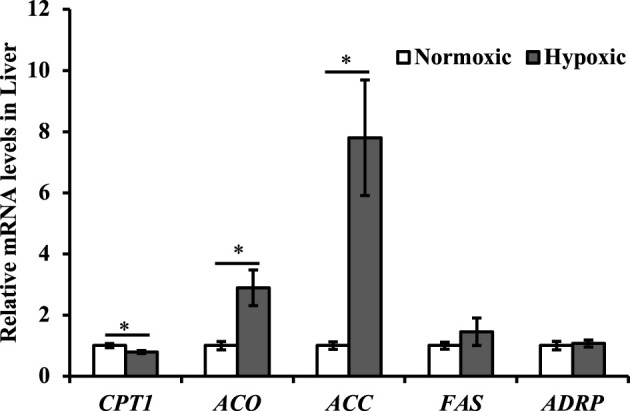
Effect of acute hypoxic stress on the expression of the lipometabolic genes in the liver of triploid rainbow trout (*n* = 6; CPT1, carnitine palmitoyl transferase 1; ACO, acetyl-CoA oxidase; ACC, acetyl-CoA carboxylase; FAS, fatty acid synthase; ADRP, adipose differentiation-related protein). Asterisks indicate significant differences (*t*-test; *p < 0.05, **p < 0.01).

Data on the mRNA expression of the lipometabolic genes are shown in Figure 6. The mRNA expression levels of *ACC* and *ACO* were upregulated, but *CPT1* was downregulated in liver tissue under hypoxic stress (*p* < 0.05). Additionally, the maximal increase in *ACC* was approximately 8-fold higher in the hypoxic group than in the normoxic group. However, no significant differences were observed in the mRNA expression levels of *FAS* and *ADRP* in liver of triploid rainbow trout under hypoxic stress (*p* > 0.05).

### Effects of Acute Hypoxic Stress on Autophagy-Related Genes Expressions in the Liver of Triploid Rainbow Trout

**FIGURE 7:**
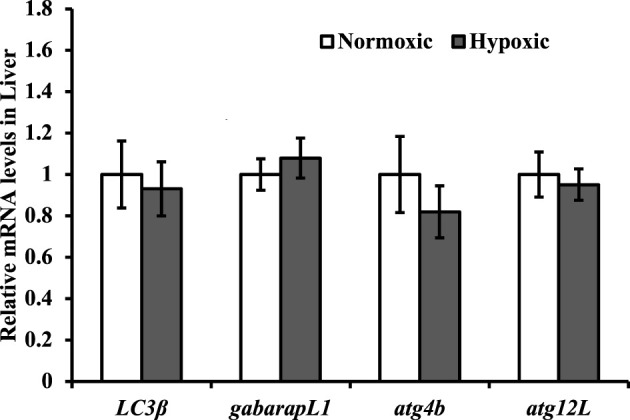
Effect of acute hypoxic stress on the expression of the autophagy-related genes in the liver of triploid rainbow trout (*n* = 6; LC3β, microtubule associated protein 1 light chain 3 beta; gabarapL1, gamma aminobutyric acid receptor-associated protein; atg4b, autophagy-related 4β; atg12L, autophagy-related 12L). Asterisks indicate significant differences (*t*-test; *p < 0.05; **p < 0.01).

No significant differences were observed in the mRNA expression levels of *LC3β*, *gabarapL1*, *atg4b*, and *atg12L* in the liver of triploid rainbow trout under hypoxic stress (*p* > 0.05; Figure 7).

## Discussion

The study simulated an acute hypoxic environment (2–5 mg/L for 10 min) caused by actual production operations of fish farming (i.e., catching, gathering, transferring, or weighting). During the hypoxic treatment, the fish firstly remained static at the bottom of the tank and then swam rapidly and moved upward to the water surface with a wide mouth opening. Finally, some fish bellied up and floated in the water. This treatment can lead to hypoxic stress in fish. When the fish body is stimulated by external adverse stress, a stress hormone, cortisol, is secreted, and its level increases in the blood (Bortoletti et al*.*, 2021). In this study, the plasma cortisol content increased under acute hypoxic stress, which could be considered a sensitive signal of fish stress (Mommsen et al*.*, 1999). A rapid, significant cortisol increase after an acute hypoxic disturbance was also found in other fish species (Abdel-Tawwab et al*.*, 2019). Positive feedback potentiates the collaboration between cortisol and glucose in rainbow trout during stress (Conde-Sieira et al., 2012). A positive correlation between cortisol and glucose levels was observed in this study. In addition, plasma ALT and AST levels were increased in the hypoxic group in this study. Particularly notable is that the ALT activity (21.8 U/l) exceeded the normal reference range (4–19 U/L) of triploid rainbow trout reported in our previous study (Meng et al., 2019), reflecting the damage to the fish’s liver. The MDA used as an indicator of lipid peroxidation and the T-AOC used as an indicator of the antioxidant capacity of the body were used in the analysis in this study. Based on the results of these indicators in the plasma and liver, the fish could experience oxidative stress under acute hypoxia.

The hypoxia-inducible factor-mediated signaling pathway has been recognized as a master regulator of cellular response to hypoxic stress (Xiao, 2015). Studies have found that acute hypoxic stress leads to upregulation of *HIF-1α* mRNA expression in the liver of fish, such as in zebrafish (*Danio rerio*) (Mandic et al., 2020), grouper (*Sebastes schlegelii*) (Jia et al., 2021), and big black mullet (*Dicentrarchus labrax*) (Vanderplancke et al., 2015). In this study, the expression of *HIF-2α* in the liver increased, but not that of *HIF-1α* under acute hypoxia stress, suggesting that *HIF-2α* is a sensitive biomarker of acute hypoxic stress in triploid rainbow trout. In mammals, HIF-2α plays a critical role in stimulating the expression of genes encoding antioxidant enzymes to suppress the accumulation of ROS, inhibiting the mitochondrial consumption of glucose-derived carbon by PDK, regulating the lipid metabolism, and improving immune responses (Reviewed in Majmundar et al., 2010). Hence, the following studies aimed to evaluate the effect of the HIF-2α signaling pathway on the antioxidant, immune, and metabolic activties of triploid rainbow trout under acute hypoxic stress.

In the antioxidant system, an increase in the expression of *Nrf2* can activate the expression of Nrf2/ARE signaling pathway-related genes, leading to an increase in antioxidant enzyme activity (Balogun et al., 2003). In this study, the expression of the *Nrf2* gene in the liver was upregulated under acute hypoxic stress, and the expression of downstream genes such as *SOD*, *CAT*, and *GPx* was suppressed or unchanged. A study on *Schizothorax prenanti* found that the expression of *SOD*, *CAT*, and *GPX* was suppressed at the initial stage of acute hypoxic stress but increased at a later stage (Zhao et al., 2020). Only 10 min of hypoxic stress was used in this study, which was too short to stimulate the genes expression of antioxidant enzymes. HO-1 is a cytoprotective enzyme that could be involved in cytoprotection and antioxidant activity against hypoxic stress (Majmundar et al., 2010; Abdel-Tawwab et al*.*, 2019). A study on rainbow trout showed that the expression of liver *HO-1* was downregulated by external adverse stress (Akdemir et al*.*, 2016). The same result was also found in this study, whereas it was opposite to the result in mammals, that is, HIF2α could activate HO-1 (Majmundar et al., 2010). Therefore, further studies are required.

Regarding the immune response to hypoxia, this study found that plasma globulin, HSP-70, and lysozyme levels increased under acute hypoxic stress. The literature has speculated that fish can increase specific proteins such as lysozyme or HSP-70 to enhance immunity level and manage stress (Ortuno et al., 2001; Ming et al*.*, 2012). In addition, HIFs can increase the production of inflammatory cytokines to participate in immunoreaction (Majmundar et al., 2010). The results of this study also showed that the expression of *IFN-γ* and *IL-1β* in the liver was upregulated after hypoxic stress. However, the expression of *TNF-α* decreased after hypoxia stress. Generally, the acute hypoxic stress made the fish body in a defensive state, thereby enhancing the immunity level in the study.

Under hypoxic stress, DO in water is insufficient to fulfill the oxygen demands for aerobic metabolism, and anaerobic metabolism is triggered (Abdel-Tawwab et al*.*, 2019). Plasma LDH activity increased sharply under acute hypoxic stress in this study, reflecting an increase in anaerobic metabolism (Ma et al., 2019). Studies have shown that the HIF-1 pathway plays a vital role in the glycometabolic switch from aerobic to anaerobic processes by regulating downstream genes, such as *GLUT* and *LDH*, and the key enzyme of glycolysis (Richards, 2009; Rademakers et al*.*, 2011; Goda and Kanai, 2014). However, the expression of *GLUT2*, *GK*, *and LDHA* in the liver did not change under acute hypoxic stress, which might be related to the lack of *HIF1a* in this study. PDK inhibits the pyruvate dehydrogenase complex and blocks the conversion of pyruvate (the glycolytic end-product) to acetyl-CoA (normally into the TCA cycle) (Mylonis et al., 2019). This study showed that the expression of *PDK1* in the liver was upregulated under acute hypoxic stress. The finding indicates that the flow of pyruvate into the mitochondria is decreased. Based on the result that the expression of *PEPCK* was upregulated in the hypoxic group, pyruvate participated in gluconeogenesis under acute hypoxic stress.

The liver is a critical site for lipid synthesis and export in fish (Tocher and Douglas, 2003). Lipids provide a rich source of energy *via* oxidative phosphorylation (Jungermann, 1988; Shohet and Garcia, 2007). Under hypoxic stress, lipid metabolism is reprogrammed to suppress lipid catabolism through β-oxidation and stimulate lipid storage and inhibition (Huss et al*.*, 2001; Bostrom et al., 2006). The regulation of metabolism is more dependent on *HIF-2a* more than *HIF-1a* (Rankin et al*.*, 2009). In this study, upregulation of the *HIF-2a* gene reduced *CPT-1* expression-mediated fatty acid mitochondrial β-oxidation and increased *ACC* expression-mediated fatty acid synthesis under acute hypoxic stress. This might explain why plasma TG levels increased in the hypoxic group. A notable result was found for *ACO*, which could control peroxisomal β-oxidation. The expression of *ACO* was upregulated under acute hypoxic stress, which might be related to the suppression of mitochondrial oxidation.

Under hypoxic conditions, autophagy is activated by ROS (Moore, 2008; Azad et al*.*, 2009), and thus serves to reduce oxidative damage (Scherz-Shouval and Elazar, 2007; Gurusamy et al*.*, 2009; Jain et al., 2015). However, the expression of autophagy-related genes (*LC3β*, *gabarapL1*, *atg4b*, and *atg12L*) in the liver was not induced by acute hypoxic stress. A possible reason for the results is that the fish body was in a defensive state after 10 min of hypoxic stress was applied.

## Conclusion

This study simulated the hypoxic environment caused by actual production operations in fish farming (i.e., catching, gathering, transferring, or weighting), which could lead to acute hypoxic stress in fish. Under the hypoxic conditions, triploid rainbow trout are in a defensive state to manage stress by enhancing immunity levels, altering antioxidant status, suppressing hepatic mitochondrial oxidation of glucose- and lipid-derived carbon, and activating hepatic gluconeogenesis and lipid synthesis. These phenotypes might be regulated by the HIF-2α pathway.

## Data Availability

The original contributions presented in the study are included in the article/Supplementary Materials, further inquiries can be directed to the corresponding author.
